# Sex-Based Differences in Imaging-Derived Body Composition and Their Association with Clinical Malnutrition in Abdominal Surgery Patients

**DOI:** 10.3390/nu18050839

**Published:** 2026-03-05

**Authors:** Raheema A. Damani, Shubha Vasisht, Valerie Luks, Gracia Vargas, Charlene Compher, Paul M. Titchenell, Gregory Tasian, Hongzhe Li, Gary D. Wu, Walter R. Witschey, Victoria M. Gershuni

**Affiliations:** 1 Department of Bioengineering, School of Engineering and Applied Sciences, University of Pennsylvania, Philadelphia, PA 19104, USA; 2 Department of Surgery, Perelman School of Medicine, University of Pennsylvania, Philadelphia, PA 19104, USA; 3 Department of Biobehavioral Health Science, School of Nursing, University of Pennsylvania, Philadelphia, PA 19104, USA; 4 Department of Clinical Nutrition Support Services, Hospital of the University of Pennsylvania, Philadelphia, PA 19104, USA; 5 Department of Physiology, Institute for Diabetes, Obesity and Metabolism, University of Pennsylvania, Philadelphia, PA 19104, USA; 6 Department of Surgery, Division of Urology, The Children’s Hospital of Philadelphia, Philadelphia, PA 19104, USA; 7 Department of Biostatistics, Epidemiology, and Informatics, University of Pennsylvania, Philadelphia, PA 19104, USA; 8 Department of Biostatistics, Epidemiology and Informatics, Perelman School of Medicine, University of Pennsylvania, Philadelphia, PA 19104, USA; 9 Division of Gastroenterology and Hepatology, Perelman School of Medicine, University of Pennsylvania, Philadelphia, PA 19104, USA; 10 Department of Radiology, Perelman School of Medicine, University of Pennsylvania, Philadelphia, PA 19104, USA

**Keywords:** sarcopenia, myosteatosis, body composition, malnutrition, nutritional risk, surgery

## Abstract

**Background**: Malnutrition significantly impacts surgical outcomes yet is difficult to identify preoperatively. Few studies have investigated the association between comprehensive body composition assessment and malnutrition in males and females separately. This study evaluates sex-specific associations between preoperative imaging-derived body composition features and malnutrition in abdominal surgery patients. **Methods**: This cross-sectional study included patients who underwent a preoperative abdominal computed tomography scan and elective abdominal surgery at a single institution (2018–2021). A deep learning algorithm quantified five muscle groups and two fat depots from CT scans. Clinical malnutrition was diagnosed by registered dietitians using standardized criteria. Sex-specific associations between imaging features and malnutrition were evaluated using logistic regression. **Results**: Among 1143 patients, 20.2% had clinical malnutrition, with prevalence varying by procedure type (3.5–38.2%). Malnutrition was associated with reduced muscle volume for both sexes; however, myosteatosis was only associated with malnutrition in females. In males, malnutrition was associated with decreased psoas volume (OR: 0.58, 95% CI [0.41–0.82]), decreased quadratus lumborum volume (OR: 0.52, 95% CI [0.35–0.77]), and reduced erector spinae attenuation (OR 0.58, 95% CI [0.41–0.82]). In females, decreased psoas volume (OR 0.56, 95% CI [0.41–0.77]) and attenuation (OR 0.59, 95% CI [0.44–0.79]) were associated with malnutrition. Both sexes showed increased subcutaneous fat attenuation (males: OR 1.58, 95% CI [1.22–2.04]; females: OR 1.96, 95% CI [1.54–2.50]) and visceral fat attenuation (males: OR 1.43 95% CI [1.07–1.90]; females: OR 1.68 95% CI [1.29–2.20]) associated with malnutrition. **Conclusions**: Males and females exhibit distinct body composition features associated with clinical malnutrition. Comprehensive analysis of muscle and fat characteristics reveals these sex-specific relationships, providing foundational knowledge for future development of predictive tools to enable earlier identification of patients at higher nutrition-related surgical risk.

## 1. Introduction

Malnutrition is an independent risk factor for poor outcomes after abdominal surgery. Surgical patients with malnutrition have increased perioperative morbidity, mortality, readmission, and length of stay [1]. Malnutrition is highly prevalent yet underrecognized, partly due to a lack of objective and easy to implement methods for nutritional assessment. While malnutrition is a modifiable risk factor, it is hard to identify in outpatient surgical clinics using current methods, which heavily rely on clinical assessment by a trained registered dietitian. Efforts to improve preoperative diagnosis of malnutrition in settings that lack a trained registered dietitian have been stymied by the lack of a reliable, automated assessment tool that can be implemented in a high-throughput manner.

Muscle mass has been highlighted as an important component of nutrition assessment and a phenotypic indicator of nutritional status according to the Global Leadership Initiative on Malnutrition criteria [2]. Muscle mass corresponds with physiologic reserve and the ability to respond to stress, including surgery. Independent of fat mass and body mass index, loss of muscle mass (sarcopenia) has been shown to be a predictor of metabolic disease, mortality [3], and adverse outcomes after surgery [4,5,6,7,8,9,10,11]. More recently, fatty replacement of muscle tissue (myosteatosis), which includes both intermuscular fat accumulation and intramyocellular lipid deposition, has been linked to impaired muscle function, disuse atrophy, aging and poor nutritional status [12,13,14,15]. Variation in intramyocellular lipid deposition has been linked to diet and exercise with increased intramyocellular lipids seen in response to prolonged starvation [16,17]. Myosteatosis is seen with insulin resistance, cardiometabolic disease, and metabolic dysfunction, independent of obesity or excess weight [18].

Body composition analysis using clinically obtained computed tomography (CT) scans provide detailed measurements of both skeletal muscle and fat stores, offering an objective method to assess nutritional status [19,20,21]. Using CT, the size and quality of multiple tissue types can be quantified, including muscle, visceral fat, and subcutaneous fat. Single-slice measures of cross-sectional area of muscle at the third lumbar vertebrae (L3) have been explored for potential to predict malnutrition in surgical populations [11,22,23]. However, a single slice may not adequately account for truncal musculature. Further, these correlations have been performed on males and females grouped together. This introduces bias, as males and females have known sex-specific differences in muscle size, deposition, and quality. Reliance on skeletal muscle area alone may underestimate risk of malnutrition in females [24,25,26]. Females have smaller muscle size and decreased muscle quality (greater fatty replacement) compared to males due to hormonal regulation via estrogen and testosterone, respectively. In addition to having less muscle at baseline, females lose muscle at a slower rate than males during periods of stress [26,27]. These sex-specific characteristics may lead to an underestimation of malnutrition risk among females when assessed using muscle size alone. 

Incorporation of more detailed body composition, including visceral and subcutaneous fat, allows for a greater understanding of the complex relationship between these metabolically active and hormone-regulated depots and the pathophysiologic state of malnutrition. Recent evidence suggests that decreased visceral fat with increased attenuation on imaging is associated with worse oncologic outcomes and worse survival in head and neck cancer, which has notoriously high rates of malnutrition [28]. Sex-specific differences in body composition, specifically visceral fat accumulation seen predominantly in males with obesity, are linked to worse cardiometabolic outcomes and inflammation [26,29]. However, the sex-specific differences between the relationship of malnutrition and hormone-mediated fat depot utilization are not clear. There is a critical gap in our understanding of the association between body composition features and nutritional status in males and females. 

This study aims to address this knowledge gap by characterizing the sex-specific associations between imaging-derived body composition features and malnutrition among males and females undergoing abdominal surgery. Here we apply a novel volumetric approach using an automated deep learning algorithm to measure five distinct muscle groups and two fat depots on clinically obtained preoperative abdominal CT scans [30,31]. The algorithm quantifies both the size and attenuation of each muscle and fat depot, offering a more comprehensive evaluation of body composition than L3 single-slice cross-sectional measures [22,23,30]. We describe the imaging features of body composition for males and females in an abdominal surgery cohort and then determine the sex-specific association between imaging features of body composition and the likelihood of diagnosis of clinical malnutrition. This foundational work establishes which body composition phenotypes co-occur with malnutrition in each sex, providing the necessary groundwork for future development of predictive risk models.

## 2. Materials and Methods

### 2.1. Study Population

We conducted a retrospective cross-sectional study that included adult patients at the Hospital of the University of Pennsylvania between 2018 and 2021 who underwent elective abdominal surgery and had a routine preoperative CT scan performed within 90 days before surgery [2]. Clinical, demographic, and outcomes data were prospectively collected for standardized inclusion in the American College of Surgeons National Surgery Quality Improvement Program (NSQIP) registry for five distinct procedures (pancreatectomy, hepatectomy, colectomy, proctectomy, and ventral hernia repair) [32]. Additional clinical, demographic, and comorbidity data, including serum albumin, weight, height, body mass index (BMI), and clinical nutrition assessment variables, were abstracted from the electronic health record, PennChart (Epic Systems Corporation, Verona, WI, USA). 

### 2.2. Nutrition Outcomes 

The primary outcome of this study was diagnosis of clinical malnutrition as determined on evaluation by a registered dietitian, which occurs after surgery. Nutritional status assessments were performed by multiple trained registered dietitians who are a part of our institution’s Clinical Nutrition Support Service; these assessments were all standardized according to the Academy ASPEN Indicators of Malnutrition (AAIM) protocols to ensure diagnostic consistency [33]. This nutrition care process includes two steps: (1) screening within 24 h of admission to the hospital using the validated Malnutrition Screening Tool [34] and (2) clinical assessment by a registered dietitian required for the formal diagnosis of clinical malnutrition. The formal assessment occurs within 24–72 h postoperatively; the timing of diagnosis is designed to explicitly capture preoperative nutritional status. The AAIM criteria include: (1) insufficient intake during the preoperative period, (2) retrospective assessment of weight loss over the preceding 1–6 months, and (3) physical examination findings of muscle and fat loss—both indicators of chronic preoperative malnutrition rather than acute postoperative changes. Patients were excluded if they experienced significant intraoperative complications or prolonged NPO status that could confound baseline nutritional assessment [33]. Patients who do not screen “At Risk” in the first step do not proceed to the formal registered dietitian assessment and are assumed to have adequate nutritional reserve. Patients with missing screening assessment were treated as having no risk for malnutrition in the primary analysis (males: *n* = 50 missing; females: *n* = 64 missing). To assess potential misclassification bias, sensitivity analyses were performed assuming a worst-case scenario where all patients with missing MST data were reclassified as malnourished. Sensitivity analyses assessed robustness to potential MST misclassification by comparing primary results to a worst-case scenario analysis (all missing MST reclassified as malnourished). Features were classified as ‘Highly Robust’ (FDR-corrected significance maintained and absolute OR change < 25%), ‘Moderately Robust’ (significance maintained but OR change ≥ 25%), or ‘Not Robust’ (significance changed after reclassification). Based on sensitivity analysis, the full cohort was included for analysis.

### 2.3. Computed Tomography Acquisition and Deep Learning-Based Image Analysis

All patients had a clinically obtained preoperative CT scan performed within 90 days prior to admission. This timeframe was selected to reflect real-world clinical practice where preoperative imaging is obtained at varying intervals based on clinical indication, scheduling, and logistical factors rather than standardized research protocols. While nutritional status can evolve during this period, particularly among patients receiving neoadjuvant therapy, body composition phenotypes such as sarcopenia and myosteatosis develop over months and provide prognostically relevant information even when assessed at varying timepoints before surgery. 

Imaging was performed using multidetector CT scanners from a variety of vendors (Philips Healthcare (Amsterdam, The Netherlands), GE Healthcare (Chicago, IL, USA), Siemens Healthineers (Erlangen, Germany), Canon Medical Systems Corporation (Otawara, Tochigi, Japan), Toshiba Medical Systems Corporation (Otawara, Tochigi, Japan)). The tube current and iodinated contrast application (including phase of contrast) varied based on the clinical setting (majority of scans acquired under portal venous contrast-enhanced (72.9%) and non-contrast (22%) protocol) (Table S3). The slice thickness varied between 2 and 5 mm. The tube voltage varied between 80 and 140 kVp (median: 100 kVp, IQR: 20 kVp), with 90.3% of scans obtained at either 100 kVp (*n* = 584, 51.1%) or 120 kVp (*n* = 448, 39.2%). The tissue types analyzed in this study—skeletal muscle and adipose tissue—demonstrate relatively stable mass attenuation coefficients across the clinical kVp range used and minimal contrast enhancement due to low vascularity, in contrast to highly vascularized organs or materials with K-edge discontinuities (iodinated contrast, calcium) which show marked sensitivity to both kVp and contrast phase [35]. Scans that were not collected in axial orientation with isotropic in-plane resolution or contained an inferior-to-superior field-of-view of less than 100 mm, or slice thickness outside of 2–5 mm range, were excluded from analysis.

Quantification of L3 single-slice measures was performed automatically using licensed software from Data Analysis Facilitation Suite version 3.6 (Voronoi Health Analytics Inc., Vancouver, BC, Canada). Previously validated L3 single-slice cross-sectional area measures included skeletal muscle index (SMI, cm^2^/m^2^) and skeletal muscle radiation attenuation (SMRA, HU) [22,36,37]. Volumetric quantification was performed using a novel deep learning approach previously described by our group [30,31]. The abdominal cavity was delineated automatically as the region between the inferior aspect of the lung (T12 vertebral level) and the inferior aspect of the L5 vertebrae. Segmentations included automated quantification of entire volume and attenuation of five individual abdominal muscle groups (psoas, erector spinae, quadratus lumborum, lateral abdominals, and rectus abdominus) and two fat depots (visceral and subcutaneous) inside CT image field-of-view. Muscle quality was determined by the attenuation of voxels contained within the muscle contour which included both muscle tissue and intermuscular adipose. Once the abdominal contour was segmented, fat voxels were identified using attenuation thresholds of −190 to −30 HU. Voxels within the abdominal contour were labeled as visceral fat, and voxels outside the abdominal contour were labeled as subcutaneous fat. All volume measurements were adjusted for patient height squared to generate a volume index (cm^3^/m^2^). Due to the novel nature of these volumetric measurements, sex-specific z-score normalization was performed using the sex-specific mean and standard deviation derived from this cohort. A list of all volumetric and single-slice imaging features can be found in Supplemental Table S1. 

### 2.4. Statistical Analysis

Descriptive statistics were used to summarize the surgical cohort stratified by sex. Continuous variables were reported as mean and standard deviation (SD) or median and interquartile range (IQR) based on distribution of data and analyzed using Student’s *t*-test or Mann–Whitney U test, respectively. Categorical variables were reported with absolute and relative frequencies and compared using Chi-square or Fisher’s exact test. Principal component analysis was used to visualize the spatial distribution of body composition features among males and females [38]. Permutational Multivariate Analysis of Variance (PERMANOVA) was used to determine if the multivariate means (group centroid) were significantly different between males compared to females [39]. The Permutation Test for Homogeneity of Multivariate Dispersion (PERMDISP) was performed to assess whether there was significant dispersion of values around the centroid for males compared to females [40].

**Figure 1:**
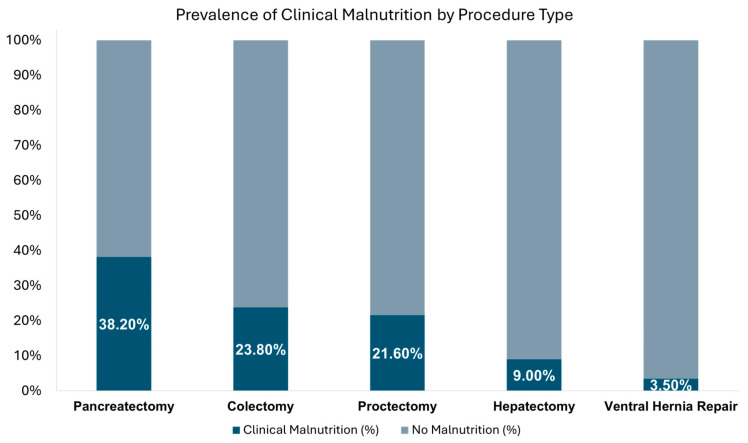
Prevalence of clinical malnutrition varies according to surgical procedure type.

Logistic regression [41] models were built to estimate the relationship between imaging-derived body composition features and clinical malnutrition. Logistic regression models were adjusted for the following covariates: age (continuous), weight (continuous), race (white vs. non-white), ASA classification (1–2 vs. 3–4), tobacco use (current smoker: yes/no), procedure type (colectomy, pancreatectomy, hepatectomy, proctectomy, or ventral hernia repair), tube voltage (kvp, continuous), and contrast phase (venous, arterial, non-contrast) [25,42]. All models were adjusted for procedure type to account for variation in baseline malnutrition prevalence across surgical procedures (Figure 1). To address multiple comparisons arising from analysis of 16 distinct imaging features, we applied the Benjamini–Hochberg False Discovery Rate (FDR) correction. All *p*-values reported in results and tables are FDR-adjusted with *p* < 0.05 indicating statistical significance. Odds ratios (OR) with 95% CI were computed for each feature for males and females separately. 

To provide clinically interpretable context for the observed associations, we performed supplemental quartile-based analyses comparing extreme quartiles (Q1 vs. Q4, with Q4 as reference) for each imaging feature in males and females separately. These quartile comparisons are descriptive and not intended as predictive cutoffs (See Supplemental Table S2). 

All statistical analyses were performed in R (R Core Team, version 4.1.2; Foundation for Statistical Computing, Vienna, Austria). A *p*-value of less than 0.05 was considered statistically significant. This study was carried out in compliance with all relevant guidelines and regulations (TRIPOD-AI) and was determined to be exempt from informed consent by the institutional review board due to the deidentified and retrospective nature of dataset.

## 3. Results

The final surgical cohort (*n* = 1143) included 48% males and 52% females. The mean age was 60.5 ± 14.6 years with an average body mass index (BMI) of 28.7 ± 7.1 kg/m^2^. A total of 75% were white and 65.7% had an ASA score greater than 3. Nearly all patients (99.1%) were functionally independent per NSQIP classification, with only 10 patients (0.9%) classified as partially or totally dependent. The most common procedure type was colectomy (504) followed by ventral hernia repair (229), pancreatectomy (204), hepatectomy (155), and proctectomy (51); 65.4% of surgeries were performed via an open approach. No clinically meaningful differences between sexes were observed for age, race, BMI, tobacco use, procedure type, operative approach, albumin, MST score, or incidence of clinical malnutrition. As expected, males had significantly higher absolute weight (86.1 vs. 73.6 kg, *p* < 0.001), reflecting expected differences in height and frame size rather than adiposity. Males were also found to have significantly higher ASA class (*p* = 0.007), prevalence of diabetes (21 vs. 14.5%, *p* = 0.008) and hypertension (49.4 vs. 43.4%, *p* = 0.04) compared to females. There were no significant differences between males and females for postoperative complications or prolonged length of stay (Table 1).

### 3.1. Prevalence of Clinical Malnutrition 

Using the Malnutrition Screening Tool upon postoperative admission to the hospital, 285 (24.9%) patients were found to be “At Risk” for malnutrition. A total of 231 patients (20.2%) were diagnosed with clinical malnutrition by a registered dietitian. Diagnosis of clinical malnutrition was highest among patients who had pancreatectomy (38.2%) followed by colectomy (23.8%), proctectomy (21.6%), hepatectomy (9.0%) and ventral hernia repair (3.5%) (Figure 1). 

### 3.2. Description of Body Composition Differences Between Males and Females 

**Figure 2:**
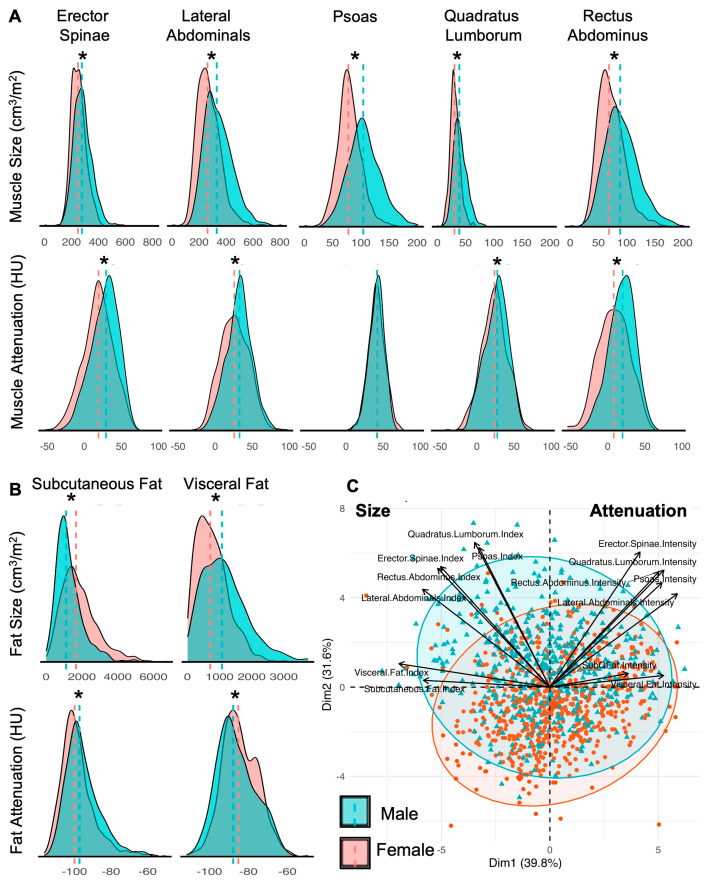
Distribution of imaging features by sex, including size (volume index, cm^3^/m^2^) and attenuation (HU) of (**A**) individual muscle groups and (**B**) fat depots. (**C**) Principal Component Analysis of body composition features cluster according to sex with significantly different centroid and dispersion. * *p* < 0.05.

Males and females had significantly different body composition using volumetric CT measures. Histograms depicting the distribution of each imaging feature are depicted in Figure 2. Males had significantly larger muscle size than females for all five muscle groups (Figure 2A: erector spinae: 282.7 ± 66.4 vs. 252.8 ± 55.1 cm^3^/m^2^, lateral abdominus: 342.3 ± 101 vs. 271.8 ± 88 cm^3^/m^2^, psoas: 106.2 ± 28.7 vs. 79.9 ± 21.2 cm^3^/m^2^, quadratus lumborum: 40.4 ± 11.8 vs. 31.3 ± 8.8 cm^3^/m^2^, rectus abdominus: 91.6 ± 30 vs. 72.6 ± 23.1 cm^3^/m^2^; all *p* < 0.001). Males displayed better muscle quality with higher attenuation for four of the five muscle groups indicating less fatty replacement of muscle tissue compared to females (Figure 2B: erector spinae: 27.3 ± 18.4 vs. 18.5 ± 20 HU, lateral abdominus: 32 ± 17 vs. 26.1 ± 19 HU, quadratus lumborum: 26 ± 16 vs. 23.4 ± 17 HU, rectus abdominus: 19 ± 17.3 vs. 8 ± 20.9 HU; all *p* < 0.001). Males had a significantly larger visceral fat depot (1156.6 ± 705 vs. 776.8 ± 493, *p* < 0.001) with lower attenuation (−86.1 ± 9.8 vs. −84.2 ± 9.1, *p* < 0.001), and they had less abdominal subcutaneous fat (1291.8 ± 755 vs. 1868 ± 1020, *p* < 0.001) with greater attenuation (−95.3 ± 9.6 vs. −98.3 ± 9.3, *p* < 0.0001) compared to females (Figure 2B). Principal component analysis (Figure 2C) demonstrated separation between males and females (*p* = 0.001). While the female class was tightly clustered, the distribution of males was more dispersed. The average distance to centroid was greater for males compared to females (3.43 vs. 3.28, *p* = 0.05), reflecting greater variability in the feature measurements.

### 3.3. Average Value of Body Composition Features Differ with Malnutrition in Males and Females 

**Figure 3:**
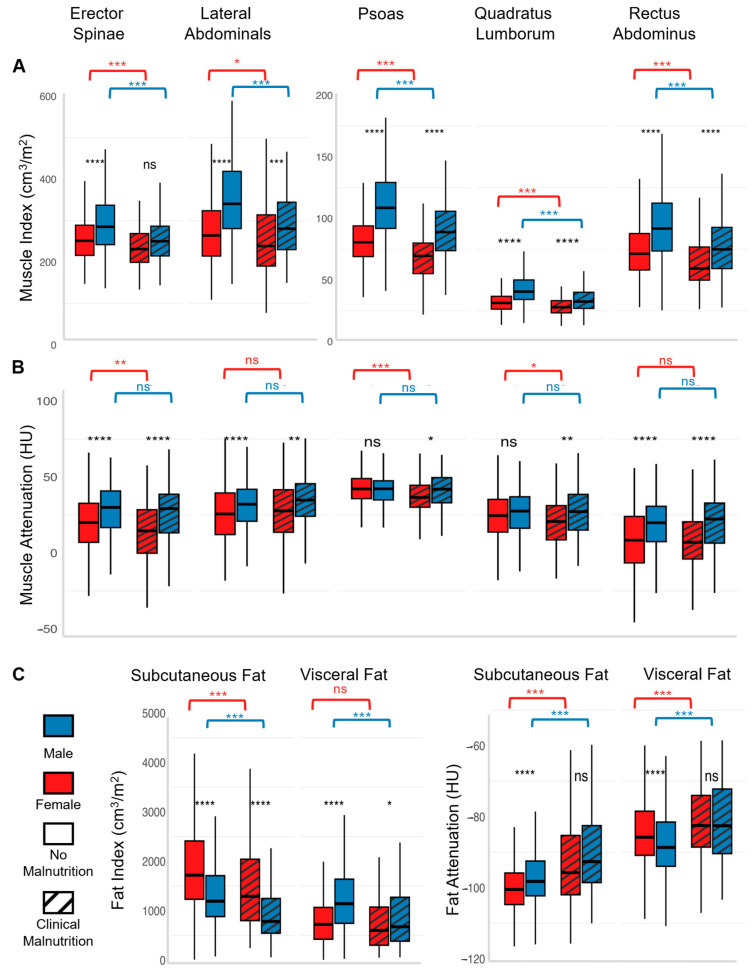
Comparison of each imaging feature: (**A**) muscle volume index, (**B**) muscle attenuation, and (**C**) fat volume index, and attenuation for males (blue) and females (red) with malnutrition (striped box) and those without (solid). Both males and females with malnutrition have smaller muscle size. Females with malnutrition have significantly decreased attenuation (fatty replacement) for three of five muscle groups. Males do not have significant differences in attenuation by nutrition status. Visceral fat stores decrease significantly in size with malnutrition for males but not for females. Visceral fat attenuation increases for both males and females with malnutrition. Both males and females with malnutrition have significantly smaller subcutaneous fat stores with greater attenuation. **** *p*<0.00001, *** *p* < 0.0001, ** *p* < 0.001, * *p* < 0.01, ns non-significant.

Both males and females with clinical malnutrition had significantly decreased size (volume index) for each individual muscle group (psoas, erector spinae, quadratus lumborum, and rectus abdominus, all *p* < 0.001, Figure 3). Females with malnutrition compared to females with normal nutrition had significantly greater fatty replacement (decreased attenuation) for three distinct muscles (erector spinae: 13.1 ± 20 vs. 19.7 ± 20 HU, *p* < 0.001, psoas: 37.7 ± 12 vs. 42 ± 11 HU, *p* < 0.0001, quadratus lumborum: 19.9 ± 19 vs. 24.2 ± 17 HU, *p* < 0.01). For males, muscle quality did not differ by nutritional status. 

Both males and females with malnutrition had a significantly smaller subcutaneous fat depot compared to those without malnutrition (Figure 3C). Males with malnutrition had significantly less visceral fat compared to males without malnutrition (869 ± 632 vs. 1236 ± 703 cm^3^/m^2^, *p* < 0.0001). While females had less visceral fat in general, there was a trend toward loss of visceral fat among females with malnutrition (704 ± 481 vs. 794 ± 495, *p* = 0.076). Both males and females had significantly increased attenuation of subcutaneous and visceral fat, suggesting alteration in size of lipid droplet or fluid content (Figure 3C).

### 3.4. Association Between Body Composition Features and Likelihood of Diagnosis of Clinical Malnutrition

Multivariable logistic regression demonstrated different relationships between individual imaging features and the odds of a diagnosis of clinical malnutrition for males and females (Table 2). For males, smaller psoas (OR 0.58 SDs [0.41 to 0.82], *p* = 0.006), smaller quadratus lumborum (OR 0.52 SDs [0.35 to 0.77], *p* = 0.005), and decreased SMI (OR 0.47 SDs [0.33 to 0.67, *p* < 0.001) were significantly associated with a greater likelihood of clinical malnutrition. For females, smaller psoas muscle volume (OR 0.56 SDs [0.41 to 0.774], *p* = 0.001) and SMI (OR 0.51 SDs [0.37 to 0.70], *p* < 0.001) were significantly associated with increased likelihood of clinical malnutrition (Table 2). 

For males, decreased attenuation (myosteatosis) of erector spinae (OR 0.58 SDs [0.41 to 0.82], *p* = 0.006), quadratus lumborum (OR 0.65 SDs, [0.45 to 0.94], *p* = 0.039), lateral abdominals (OR 0.61 SDs [0.43 to 0.88], *p* = 0.017), and L3 skeletal muscle radiation attenuation (OR 0.56 SDs, [0.39 to 0.81], *p* = 0.006) was associated with greater risk of malnutrition (Table 2B). For females, decreased attenuation of psoas muscle (OR 0.59 SDs [0.44 to 0.79], *p* = 0.001), erector spinae muscle (OR 0.58 [0.42 to 0.81], *p* = 0.003), quadratus lumborum (OR 0.66 SDs, [0.48 to 0.93], *p* = 0.031), and L3 skeletal muscle radiation attenuation (OR 0.57 SD [0.41 to 0.79], *p* = 0.002) were associated with greater odds of malnutrition. For both males and females, increased subcutaneous fat attenuation (OR 1.58 SDs [1.22 to 2.04], *p* = 0.004 and OR 1.96 SDs [1.54 to 2.50], *p* < 0.001) and increased visceral fat attenuation (OR 1.43 SDs [1.07–1.90], *p* = 0.029 and OR 1.68 SDs [1.29–2.02], *p* = 0.001, respectively) were both associated with greater odds of malnutrition.

Quartile-based analyses (Supplemental Table S2) demonstrated the clinical magnitude of associations between body composition features and malnutrition. In males, comparing lowest to highest quartiles, psoas volume showed OR 0.17 [95% CI 0.06–0.47] (*p* = 0.001) and SMI showed OR 0.25 [0.11–0.58] (*p* = 0.001). In females, psoas volume showed OR 0.26 [0.11–0.60] (*p* = 0.01), SMI showed OR 0.28 [0.13–0.62], (*p* = 0.01), psoas attenuation showed OR 0.26 [0.12–0.55] (*p* < 0.001), and subcutaneous fat attenuation showed OR 3.81 [1.93–7.55] (*p* < 0.001). These quartile ranges are specific to our cohort and presented for descriptive purposes; they are not validated clinical thresholds.

### 3.5. Sensitivity to Missing Data

Sex-stratified sensitivity analyses demonstrated that primary findings were robust to potential MST misclassification. Under worst-case scenarios where all patients with missing MST were reclassified as malnourished (males: 50 additional cases, increasing prevalence from 21.7% to 30.9%; females: 64 additional cases, increasing prevalence from 18.9% to 29.6%), the majority of key imaging features maintained statistical significance with OR changes < 25% (Supplemental Tables S4A,B).

In males, highly robust features included psoas volume (19.7% OR change), quadratus lumborum volume (20.2%), muscle attenuation measures (15–21%), and subcutaneous fat attenuation (16.5%). In females, highly robust features included muscle attenuation measures (18.5–20.4% OR change) and subcutaneous fat attenuation (20.2%). Moderately robust features included SMI in both sexes (males: 27.7% OR change; females: 25.1%) and psoas volume in females (28.5%). Features showing significance changes under worst-case assumptions (lateral abdominal attenuation in males; quadratus lumborum attenuation, visceral fat attenuation, and subcutaneous fat index and attenuation in females) should be considered exploratory findings requiring validation.

## 4. Discussion

This study identifies preoperative imaging-derived body composition features associated with clinical malnutrition among males and females undergoing major abdominal surgery. Our findings demonstrate that body composition analysis using routine preoperative CT scans can identify various characteristics (imaging-derived features) associated with malnutrition. Further, while both males and females with malnutrition show reduced muscle size, the patterns of muscle quality deterioration (myosteatosis) and fat distribution vary significantly between sexes, suggesting that accounting for patient sex and multiple muscle and fat metrics may be necessary for accurate nutritional assessment using imaging-based body composition analysis.

Several key findings emerge from our analysis. First, in keeping with the literature, we observed that males and females exhibit distinct body composition profiles independent of nutritional status, with males showing greater volumes of both muscle and visceral fat, while females have smaller muscle with more subcutaneous fat deposition. Both males and females with malnutrition exhibited significantly smaller muscle volumes compared to those without malnutrition, consistent with known associations between sarcopenia and malnutrition [43]. Importantly, females with normal nutritional status had similar muscle size compared to males with malnutrition, which highlights the discrepancy in size between the sexes and the importance of sex-disaggregated analysis. The relationship between malnutrition risk and decreased muscle size—both psoas volume index and L3 SMI—was true for both sexes. However, the association between other muscle features and malnutrition for males and females separately suggest that a more nuanced approach using comprehensive volumetric analysis is needed to develop imaging biomarkers and for future use in predictive risk models.

Second, we found that females with malnutrition had significantly decreased mean muscle attenuation for three out of the five muscle groups compared to females without malnutrition. For males, however, there was no significant difference in mean muscle attenuation for males with or without malnutrition. Decreased muscle attenuation is seen with myosteatosis and represents decreased muscle quality. Myosteatosis includes both intramuscular adipose deposition and intramyocellular lipid droplet accumulation. Intramuscular adipose tissue has been linked to impaired skeletal muscle function with decreased strength and metabolic disease, including insulin resistance [44,45]. Using logistic regression, we determined that differences in muscle attenuation were valuable for determining likelihood of malnutrition in both males and females. In a recently published study, Xie et al. also found sex-specific differences in myosteatosis among older hospitalized females with malnutrition. This relationship was not significant for males. Together, these similar findings suggest that fatty deposition and change in muscle quality are more sensitive imaging characteristics for females with malnutrition [46]. Increased fatty replacement, specifically intramuscular adipose tissue, has been associated with decreased force generation and worse function, especially among those with metabolic disease [44]. Similarly, myosteatosis is associated with worse outcomes after surgery [47,48,49]. For females, who inherently have smaller muscle size, increased fatty replacement may be a more significant marker of decreased muscle function as seen in sarcopenia and frailty. These imaging findings are clinically meaningful; in a meta-analysis of 19 papers, preoperative myosteatosis has been shown to be associated with poor outcomes and decreased overall survival after surgery for gastrointestinal malignancy [50]. These findings highlight the value of incorporating muscle quality in addition to muscle size as an indicator of malnutrition, especially for female patients.

Our findings regarding fat depletion and change in attenuation are particularly noteworthy. Males are known to have greater visceral fat deposition due to hormonal influences [25,26,51]. Here we demonstrate an association between clinical malnutrition and loss of visceral fat with increased attenuation, which has the potential to be an imaging-based biomarker for inadequate nutrition. CT findings demonstrate both loss of stored fat and decreased size of lipid droplet possibly secondary to increased lipolysis and enhanced fatty acid uptake which occurs as a compensatory response to inadequate nutrition [14,52,53,54]. Adipocyte lipolysis is regulated by multiple hormonal cues that including insulin and catecholamines known to be aberrant in malnutrition. The amount of visceral fat was not significantly different for females with and without malnutrition. However, increased mean attenuation of both subcutaneous and visceral fat, which may represent lipolysis and fat mobilization, was associated with greater likelihood of malnutrition. In non-human primates and in studies of cancer cachexia, increased adipose attenuation has been linked to increased mortality [55]. Change in adipose tissue attenuation is thought to reflect adipose remodeling, including decreased fat cell volume with increased fibrosis of surrounding connective tissue [56,57]. This imaging characteristic of increased adipose attenuation has been associated with deteriorating health and is an imaging marker of former obesity, both in weight loss associated with aging and as seen in cancer cachexia [55,57,58,59,60]. Both males and females with malnutrition had increased attenuation of visceral and subcutaneous fat suggesting that after depletion of stored lipids, lipocytes reach a similar minimum size in both males and females. These imaging-based findings have important implications for nutritional assessment protocols, can serve as imaging biomarkers for underlying physiologic response to inadequate nutrition, and will improve our ability to identify malnutrition in a high-throughput and reliable way.

These results have several important clinical implications. First, they suggest that nutritional assessment tools incorporating body composition analysis should account for sex-specific differences in both muscle and fat characteristics. Second, the finding that muscle quality is particularly important in females indicates that evaluation of muscle mass alone may be insufficient for accurate nutritional assessment. Quartile-based analyses highlight the clinical significance of these findings: males in the lowest quartile of psoas volume and females in the lowest quartile of psoas attenuation (highest myosteatosis) had 6-fold and 4-fold higher odds of malnutrition, respectively. These substantial effect sizes suggest that body composition features capture meaningful nutritional vulnerability. However, as these descriptive ranges are derived from a single-institution cohort, they require prospective validation in independent populations before application as diagnostic thresholds. Third, the comprehensive volumetric analysis approach used in this study provides a more thorough assessment than traditional single-slice measurements and supports the development of image-based screening models to identify patients with malnutrition in the preoperative setting, presenting an opportunity for preoperative nutritional optimization.

Our sex-stratified sensitivity analyses addressing potential misclassification from missing MST data demonstrated that primary findings are robust even under worst-case assumptions. In both sexes, the majority of key features remained significant with OR changes < 25% when all missing cases were assumed malnourished. Notably, the sex-specific patterns we identified—muscle volume measures in males, muscle quality (attenuation) in females, and subcutaneous fat attenuation in both sexes—all demonstrated high to moderate robustness. Features showing significance changes (lateral abdominal attenuation in males; quadratus lumborum and visceral fat attenuation in females) should be considered exploratory, reinforcing the importance of our tiered analytical approach and the need for validation of secondary findings.

Several limitations should be considered when interpreting these results. First, the retrospective nature of the study and single-institution setting may limit generalizability. Second, while we adjusted for procedure type in all models and malnutrition prevalence varied substantially across procedures (Figure 1), small sample sizes within individual procedure subgroups precluded robust procedure-specific or cancer-stratified analyses. Future multi-center studies with larger procedure-specific cohorts will be needed to determine whether body composition–malnutrition associations differ meaningfully across surgical contexts or by cancer diagnosis. Third, the use of clinical malnutrition diagnosis as the reference standard, while pragmatic, may be subjective despite standardized criteria. We acknowledge that malnutrition assessment occurred postoperatively; however, this reflects the current standard of care at most institutions, where comprehensive nutritional screening and dietitian assessment are triggered by hospital admission rather than during outpatient preoperative visits. While it would be ideal to assess malnutrition preoperatively, this is not pragmatically feasible in most clinical settings given resource constraints and the lack of routine registered dietitian access in outpatient surgical clinics. Importantly, the AAIM diagnostic criteria specifically capture chronic preoperative nutritional depletion through retrospective weight history and physical findings indicative of longstanding muscle/fat loss rather than acute surgical effects. Our study design mirrors real-world clinical practice and demonstrates proof-of-concept that preoperative imaging could complement—and potentially anticipate—postoperative nutritional assessment, enabling earlier identification of at-risk patients when intervention opportunities are greatest. Fourth, CT protocol heterogeneity may have introduced measurement variability in attenuation; however, several factors substantially mitigate this concern: (1) the tissue types measured (muscle and fat) have relatively stable mass attenuation coefficients across clinical kVp ranges and demonstrate minimal contrast enhancement due to low vascularity, unlike highly vascularized organs or materials with K-edge discontinuities (iodine, calcium); (2) the majority of scans were obtained using similar protocols (90% at 100–120 kVp, 73% portal venous phase); and (3) our analyses focus on associations between HU measurements and malnutrition rather than defining absolute HU thresholds. While this heterogeneity reflects real-world clinical practice and enhances generalizability, future studies with standardized imaging protocols would further reduce measurement variability. Finally, the cross-sectional nature of our analysis prevents determination of causality in the relationship between body composition changes and malnutrition.

Future research should prioritize validating these findings through prospective, multi-center studies to derive and validate sex-specific body composition thresholds in larger, diverse cohorts. Beyond individual metrics, there is clear need to develop integrative predictive models that combine multiple imaging features to enhance diagnostic precision. Additionally, prospective studies are required to determine the optimal sensitivity and specificity trade-offs for clinical decision making, ensuring these tools are both practical and reliable in a surgical setting. Finally, investigating longitudinal changes in body composition features during nutritional intervention may provide deeper insights into sex-specific responses to nutritional support and their subsequent impact on postoperative outcomes. 

## 5. Conclusions

This study demonstrates significant sex-specific associations between body composition features on preoperative imaging and malnutrition among males and females undergoing abdominal surgery. This descriptive analysis establishes which muscle, and fat characteristics differ between patients with and without malnutrition, revealing distinct patterns by sex. While males and females with malnutrition both show reduced muscle size, the patterns of muscle quality deterioration (myosteatosis) and fat distribution vary significantly between sexes, suggesting that accounting for patient sex and multiple muscle and fat metrics may be necessary for accurate nutritional assessment using imaging-based body composition analysis.

These findings provide critical foundational knowledge for the next step: developing and validating sex-specific predictive models that can prospectively identify patients at risk for malnutrition using preoperative imaging. Such predictive tools could leverage CT scans already performed for surgical planning to enable automated preoperative risk stratification without requiring additional testing or registered dietitian access in outpatient settings. Future work should focus on building these predictive models, validating them in independent cohorts, and testing their clinical utility in prospective implementation studies. Understanding these sex-specific body composition–malnutrition associations is an essential first step toward more precise and personalized approaches to preoperative nutritional assessment and intervention strategies. 

## Figures and Tables

**Table 1:** Demographic and clinical characteristics for abdominal surgery patients by sex.

Characteristic	Total (*n* = 1143)	Male (*n* = 544)	Female (*n* = 599)	*p*-Value
Age (years)	60.51 ± 14.64	60.9 ± 14.8	60.9 ± 15	0.931
*Race*				
White	856 (74.9%)	419 (77%)	437 (73.3%)	0.430
Black	204 (17.8%)	88 (16.2%)	116 (19.4%)	
Other	83 (7.3%)	37 (6.8%)	46 (7.7%)	
Weight (kg)	82.5 + 21.9	86.1 ± 19	73.6 ± 19.8	0.001
BMI (kg/m^2^)	28.7 ± 7.08	27.3 ± 5.4	28 ± 7.3	0.191
At Risk for Malnutrition (MST)	285 (24.9%)	134 (24.6%)	151 (25.2%)	0.695
Clinical Malnutrition	231 (20.2%)	118 (21.7%)	113 (18.9%)	0.265
Albumin < 3.5 g/dL	172 (15.04%)	84 (15.4%)	88 (14.7%)	0.786
*ASA Classification*				
1–2	392 (34.3%)	165 (30.3%)	227 (37.9%)	0.007
3–4	751 (65.7%)	379 (69.7%)	372 (62.1%)	
Current Smoker	127 (11.1%)	65 (11.9%)	62 (10.4%)	0.445
*Diabetes Mellitus*				
Insulin	89 (7.78%)	53 (9.7%)	36 (6%)	0.008
Non-Insulin	115 (10.1%)	64 (11.8%)	51 (8.5%)	
No	939 (82.2%)	427 (78.5%)	512 (85.5%)	
Hypertension	529 (46.3%)	269 (49.4%)	260 (43.4%)	0.044
Disseminated Cancer	144 (12.6%)	70 (12.9%)	74 (12.4%)	0.863
*Procedure Type*				
Colectomy	504 (44.1%)	238 (43.8%)	266 (44.4%)	0.225
VHR	229 (20%)	105 (19.3%)	124 (20.7%)	
Pancreatectomy	204 (17.8%)	91 (16.7%)	113 (18.9%)	
Hepatectomy	155 (13.6%)	87 (16%)	68 (11.4%)	
Proctectomy	51 (4.46%)	23 (4.2%)	28 (4.7%)	
*Operative Approach*				
Laparoscopic	317 (27.7%)	141 (25.9%)	176 (29.4%)	0.718
Lap converted to Open	79 (6.9%)	40 (7.35%)	39 (6.5%)	
Open	747 (65.4%)	363 (66.7%)	384 (64.1%)	
*Surgical Outcomes*				
Any Complication	224 (19.6%)	113 (20.7%)	111 (18.5%)	0.380
Long Length of Stay	256 (22.4%)	127 (23.3%)	129 (21.5%)	0.435

ASA: American Society of Anesthesiologists; MST: Malnutrition Screening Tool; Mean ± standard deviation is reported for continuous features. The frequency and percentage of patients in each category is reported for categorical features.

**Table 2:** Association between imaging-derived features and likelihood of clinical malnutrition for males and females using logistic regression adjusted for age, weight, race, ASA classification, tobacco use, procedure type, kVp, and contrast phase. All *p*-values are FDR-adjusted (q < 0.05) to control for multiple comparisons across 16 imaging features.

(A) Association between imaging-derived feature (size) and likelihood of clinical malnutrition.				
	Males		Females	
Imaging Feature: Size	Odds Ratio [95%CI]	*p*-Value	Odds Ratio [95%CI]	*p*-Value
Muscle—Volume Index (cm^3^/m^2^)				
Psoas	0.58 [0.41–0.82]	0.006	0.56 (0.41–0.77)	0.001
Erector Spinae	0.69 [0.48–0.98]	0.059	1.03 (0.77–1.38)	0.907
Quadratus Lumborum	0.52 [0.35–0.77]	0.005	0.87 (0.62–1.21)	0.538
Lateral Abdominals	0.82 [0.58–1.16]	0.291	1.37 (1.00–1.89)	0.089
Rectus Abdominus	0.74 [0.52–1.06]	0.133	1.00 (0.71–1.40)	0.986
Fat—Volume Index (cm^3^/m^2^)				
Subcutaneous Fat	0.90 (0.54–1.49)	0.682	0.70 (0.41–1.18)	0.286
Visceral Fat	0.90 (0.61–1.32)	0.626	1.12 (0.79–1.58)	0.643
L3 Single Slice (cm^2^/m^2^)				
Skeletal Muscle Index	0.47 (0.33–0.67)	<0.001	0.51 (0.37–0.70)	<0.001
(B) Association between imaging-derived feature (attenuation) and likelihood of clinical malnutrition				
	Males		Females	
Imaging Feature: Attenuation	Odds Ratio [95%CI]	*p*-Value	Odds Ratio [95%CI]	*p*-Value
Muscle—Attenuation (HU)				
Psoas	0.71 (0.51–0.99)	0.059	0.59 (0.44–0.79)	0.001
Erector Spinae	0.58 (0.41–0.82)	0.006	0.58 (0.42–0.81)	0.003
Quadratus Lumborum	0.65 (0.45–0.94)	0.039	0.66 (0.48–0.93)	0.031
Lateral Abdominals	0.61 (0.43–0.88)	0.017	0.86 (0.62–1.22)	0.538
Rectus Abdominus	0.79 (0.57–1.10)	0.198	0.92 (0.68–1.25)	0.681
Fat—Attenuation (HU)				
Subcutaneous Fat	1.58 (1.22–2.04)	0.004	1.96 (1.54–2.50)	0.000
Visceral Fat	1.43 (1.07–1.90)	0.029	1.68 (1.29–2.20)	0.001
L3 Single Slice (HU)				
Skeletal Muscle Attenuation	0.56 (0.39–0.81)	0.006	0.57 (0.41–0.79)	0.002

## Data Availability

The original contributions presented in this study are included in the article/Supplementary Materials. Further inquiries can be directed to the corresponding author.

## Supplementary Materials

The following supporting information can be downloaded at: https://www.mdpi.com/article/10.3390/nu18050839/s1, Table S1: List of imaging-derived body composition features; Table S2: Quartile-based analysis comparing lowest quartile (Q1) to highest quartile (Q4, reference group) for males; Table S3: Prevalence (N (%)) of CT scan parameters in patients with clinical malnutrition vs. not malnourished group in our cohort; Table S4: Sensitivity analysis comparing primary analysis (missing MST = no risk) to worst-case scenario (all missing MST reclassified as malnourished).

## Abbreviations

The following abbreviations are used in this manuscript:

BMI	Body Mass Index
CT	Computed Tomography
HU	Hounsfield Units
SMI	Skeletal Muscle Index
SMRA	Skeletal Muscle Radiation Attenuation
L3	3rd Lumbar Vertebrae
AAIM	Academy ASPEN Indicators of Malnutrition
NSQIP	National Surgical Quality Improvement Program
OR	Odds Ratio
