# Supplementary material for: Sex-Based Differences in Imaging-Derived Body Composition and Their Association with Clinical Malnutrition in Abdominal Surgery Patients
Source: Nutrients. 2026 Mar 5;18(5):839. doi: 10.3390/nu18050839 (PMC12986700; doi:10.3390/nu18050839)
Supplement: Supplementary file 1 [file nutrients-18-00839-s001.zip › nutrients-4134115-supplementary.pdf]

**Supplemental Table S1. List of imaging-derived body composition features**

| | Size Feature | Attenuation Feature (HU) |
|---|---|---|
| 2D | L3 Skeletal Muscle Index ($cm^2/m^2$) | L3 Skeletal Muscle Radiation Attenuation |
| 3D | Psoas Index ($cm^3/m^2$) | Psoas Attenuation |
| 3D | Quadratus Lumborum Index ($cm^3/m^2$) | Quadratus Lumborum Attenuation |
| 3D | Erector Spinae Index ($cm^3/m^2$) | Erector Spinae Attenuation |
| 3D | Rectus Abdominus Index ($cm^3/m^2$) | Rectus Abdominus Attenuation |
| 3D | Lateral Abdominals Index ($cm^3/m^2$) | Lateral Abdominals Attenuation |
| 3D | Visceral Fat Index ($cm^3/m^2$) | Visceral Fat Attenuation |
| 3D | Subcutaneous Fat Index ($cm^3/m^2$) | Subcutaneous Fat Attenuation |

**Supplementary Table S2**: Quartile-based analysis comparing lowest quartile (Q1) to highest quartile (Q4, reference group) for males. Adjusted for age, race, weight, smoking, ASA classification, and procedure type. Bold p-values indicate significance after FDR correction. **Quartile ranges are specific to this cohort and presented for descriptive purposes only—they are not validated clinical thresholds.**

**Supplementary Table S2a:** Association between imaging derived feature (size) and likelihood of clinical malnutrition - Quartile Analysis

| | Males | | | | Females | | | |
|---|---|---|---|---|---|---|---|---|
| Imaging Feature: Size | Q1 range | Q4 range | Odds Ratio [95%CI] | *p-value* | *Q1 range* | *Q4 range* | Odds Ratio [95%CI] | *p-*value |
| Muscle - Volume Index ($cm^3/m^2$) | | | | | | | | |
| Psoas | 37.8-87.9 | 124.0-196.5 | 0.17 [0.06-0.47] | **0.02** | 21.9-67.1 | 91.3-197.0 | 0.25 [0.11-0.59] | **0.01** |
| Erector Spinae | 98.1-235.6 | 321.5-566.3 | 0.58 [0.24-1.40] | 0.34 | 134-212.5 | 285.2-578.8 | 0.85 [0.41-1.77] | 0.87 |
| Quadratus Lumborum | 10.0-32.1 | 47.7-84.4 | 0.32 [0.12-0.83] | 0.11 | 10.0-25.8 | 36.3-64.7 | 1.03 [0.47-2.23] | 0.95 |
| Lateral Abdominals | 146.9-267.6 | 407.8-709.1 | 0.56 [0.25-1.26] | 0.28 | 77.4-207.3 | 321.3-809.6 | 1.36 [0.63-2.96] | 0.63 |
| Rectus Abdominus | 25.3-71.5 | 109.9-201.9 | 0.73 [0.31-1.70] | 0.54 | 26.3-56.3 | 85.8-167.0 | 0.82 [0.37-1.82] | 0.87 |
| Fat - Volume Index ($cm^3/m^2$) | | | | | | | | |
| Subcutaneous Fat | 58.0-771.4 | 1622-5021 | 0.91 [0.34-2.39] | 0.87 | 208-1144.9 | 2343-6744 | 0.44 [0.15-1.26] | 0.30 |
| Visceral Fat | 27.3-606.6 | 1533-3758 | 1.00 [0.41-2.48] | 0.99 | 50.1-400.0 | 1069-3003 | 1.07 [0.48-2.40] | 0.88 |
| L3 Single Slice ($cm^2/m^2$) | | | | | | | | |
| Skeletal Muscle Index | 18.9-35.8 | 48.2-83.2 | 0.25 [0.11-0.58] | **0.02** | 16.7-29.9 | 39.0-66.3 | 0.27 [0.12-0.59] | **0.01** |

**Supplementary Table S2b:** Association between imaging derived feature (attenuation) and likelihood of clinical malnutrition - Quartile Analysis

| | Males | | | | Females | | | |
|---|---|---|---|---|---|---|---|---|
| Imaging Feature: Attenuation | Q1 range | Q4 range | Odds Ratio [95%CI] | *p*-value | *Q1 range* | *Q4 range* | Odds Ratio [95%CI] | *p*-value |
| Muscle - Attenuation (HU) | | | | | | | | |
| Psoas | 10.3-34.3 | 48.0-65.6 | 0.70 [0.33-1.46] | 0.46 | 4.5-33.9 | 48.2-74.3 | 0.26 [0.12-0.55] | **0.005** |
| Erector Spinae | -47.7 - 15.5 | 40.2-68.1 | 0.38 [0.15-0.92] | 0.15 | -45.6-6 | 31.4-66.0 | 0.43 [0.18-1.01] | 0.16 |
| Quadratus Lumborum | -23.6 - 15.9 | 37.3-65.7 | 0.67 [0.29-1.57] | 0.46 | -39.1-11.6 | 34.2-70.7 | 0.38 [0.16-0.89] | 0.13 |
| Lateral Abdominals | -16.3 - 21.3 | 42.6-78.5 | 0.49 [0.21-1.16] | 0.26 | -26.8-12.2 | 39.6-97.3 | 0.91 [0.41-2.06] | 0.88 |
| Rectus Abdominus | -31.4-7.2 | 31.3-61.4 | 0.75 [0.34-1.65] | 0.54 | -54.3-(-6.7) | 23.4-55.8 | 0.84 [0.37-1.90] | 0.85 |
| Fat - Attenuation (HU) | | | | | | | | |
| Subcutaneous Fat | -116-(-101.9) | -90.6-(-50.2) | 2.51 [1.22-5.16] | 0.10 | -116.5- (-104.2) | -94.8-(-55.9) | 3.81 [1.93-7.55] | **0.005** |
| Visceral Fat | -110.8- (-93.2) | -79.8-(-58.5) | 1.59 [0.75-3.36] | 0.34 | -111.8- (-90.6) | -77.2 -(-49.5) | 1.95 [1.02-3.72] | 0.16 |
| L3 Single Slice (HU) | | | | | | | | |
| Skeletal Muscle Attenuation | 6.2-27.3 | 43.9-64.0 | 0.35 [0.15-0.82] | 0.10 | -2.3-24.2 | 42.0-68.2 | 0.41 [0.18-0.92] | 0.13 |

**Supplementary Table S3:** Prevalence (N (%)) of CT scan parameters in patients with clinical malnutrition vs. not malnourished group in our cohort

| Scan Parameters | Total (N=1143, (%)) | Not Malnourished (N=912, (%)) | Clinical Malnutrition (N=231, (%)) |
|---|---|---|---|
| Contrast Phase | | | |
| portal venous | 833 (72.9) | 668 (73.2) | 165 (71.4) |
| non-contrast | 251 (21.9) | 199 (21.8) | 52 (22.5) |
| late arterial | 54 (4.7) | 42 (3.7) | 12 (5.2) |
| early arterial | 5 (0.4) | 3 (0.3) | 2 (0.9) |
| Tube Voltage (kVP) | | | |
| 80-90 | 26 (2.3) | 14 (1.5) | 12 (5.2) |
| 100-120 | 1055 (92.3) | 848 (92.9) | 207 (89.6) |
| 130-140 | 62 (5.4) | 50 (5.5) | 12 (5.2) |

**Supplementary Table S4**: Sensitivity analysis comparing primary analysis (missing MST = no risk) to worst-case scenario (all missing MST reclassified as malnourished). 'Highly Robust' = FDR-corrected significance maintained with <25% OR change. 'Moderately Robust' = significance maintained with ≥25% OR change. 'Not Robust' = significance changed.

Supplementary Table S4A: Sensitivity analysis reclassifying missing MST scores for males as malnourished (N=50) and assessing association of imaging features with clinical malnutrition

| | Missing as Not Malnourished | | Missing as Malnourished | | | |
|---|---|---|---|---|---|---|
| Imaging Feature | OR [95% CI] | p-value | OR [95% CI] | p-value | % Change in OR | Robustness |
| **SMI** | 0.491 [0.35-0.69] | <0.001 | 0.627 [0.48-0.82] | 0.001 | 27.7 | **Moderately Robust** |
| **SMRA** | 0.607 [0.43-0.85] | 0.004 | 0.737 [0.56-0.97] | 0.028 | 21.4 | **Highly Robust** |
| **Psoas Index** | 0.609 [0.43-0.85] | 0.004 | 0.729 [0.56-0.95] | 0.021 | 19.7 | **Highly Robust** |
| **Erector Spinae Index** | 0.71 [0.50-1.01] | 0.056 | 0.801 [0.61-1.06] | 0.116 | 12.8 | **Highly Robust** |
| **Quadratus Lumborum Index** | 0.534 [0.36-0.79 | 0.001 | 0.642 [0.47-0.87] | 0.004 | 20.2 | **Highly Robust** |
| **Lateral Abdominals Index** | 0.843 [0.60-1.19 | 0.329 | 0.877 [0.66-1.16] | 0.356 | 4 | **Highly Robust** |
| **Rectus Abdominus Index** | 0.772 [0.55-1.09] | 0.144 | 0.885 [0.67-1.17] | 0.385 | 14.6 | **Highly Robust** |
| **Psoas Attenuation** | 0.758 [0.57-1.01] | 0.057 | 0.829 [0.65-1.06] | 0.129 | 9.4 | **Highly Robust** |
| **Erector Spinae Attenuation** | 0.613 [0.44-0.85] | 0.004 | 0.705 [0.54-0.93] | 0.013 | 15 | **Highly Robust** |
| Quadratus Lumborum Attenuation | 0.693 [0.49-0.97] | 0.033 | 0.777 [0.59-1.03] | 0.076 | 12.1 | Not Robust |
| Lateral Abdominals Attenuation | 0.637 [0.46-0.89] | 0.008 | 0.762 [0.58-1.00] | 0.054 | 19.6 | Not Robust |
| **Rectus Abdominus Attenuation** | 0.807 [0.60-1.08] | 0.156 | 0.909 [0.71-1.17] | 0.456 | 12.6 | **Highly Robust** |
| **Subcutaneous Fat Index** | 0.948 [0.58-1.55] | 0.833 | 0.848 [0.58-1.23] | 0.384 | -10.5 | **Highly Robust** |
| **Visceral Fat Index** | 0.933 [0.64-1.36] | 0.72 | 0.981 [0.72-1.34] | 0.901 | 5.1 | **Highly Robust** |
| **Subcutaneous Fat Attenuation** | 1.55 [1.21-1.99] | 0.001 | 1.294 [1.05-1.60] | 0.018 | -16.5 | **Highly Robust** |
| Visceral Fat Attenuation | 1.363 [1.04-1.78] | 0.023 | 1.126 [0.90-1.41] | 0.299 | -17.4 | Not Robust |

Supplementary Table S4B: Sensitivity analysis reclassifying missing MST scores for females as malnourished (N=64) and assessing association of imaging features with clinical malnutrition

| | Missing as Not Malnourished | | Missing as Malnourished | | | |
|---|---|---|---|---|---|---|
| Imaging Feature | OR [95% CI] | p-value | OR [95% CI] | p-value | % Change in OR | Robustness |

| | | | | | | |
|---|---|---|---|---|---|---|
| SMI | 0.513 [0.37-0.70] | <0.001 | 0.642 [0.50-0.82] | <0.001 | 25.1 | Moderately Robust |
| SMRA | 0.594 [0.43-0.81] | 0.001 | 0.704 [0.54-0.91] | 0.008 | 18.5 | Highly Robust |
| Psoas Index | 0.551 [0.40-0.75] | <0.001 | 0.708 [0.56-0.90] | 0.005 | 28.5 | Moderately Robust |
| Erector Spinae Index | 0.993 [0.74-1.33] | 0.963 | 0.915 [0.72-1.16] | 0.457 | -7.9 | Highly Robust |
| Quadratus Lumborum Index | 0.852 [0.61-1.19] | 0.341 | 0.851 [0.66-1.10] | 0.222 | -0.1 | Highly Robust |
| Lateral Abdominals Index | 1.317 [0.96-1.80] | 0.085 | 1.102 [0.85-1.43] | 0.462 | -16.3 | Highly Robust |
| Rectus Abdominus Index | 0.955 [0.69-1.33] | 0.785 | 0.936 [0.72-1.21] | 0.614 | -2 | Highly Robust |
| Psoas Attenuation | 0.623 [0.48-0.81] | <0.001 | 0.75 [0.60-0.94] | 0.014 | 20.4 | Highly Robust |
| Erector Spinae Attenuation | 0.582 [0.42-0.80] | 0.001 | 0.698 [0.53-0.91] | 0.009 | 19.9 | Highly Robust |
| Quadratus Lumborum Attenuation | 0.674 [0.49-0.92] | 0.013 | 0.808 [0.62-1.05] | 0.116 | 19.9 | Not Robust |
| Lateral Abdominals Attenuation | 0.856 [0.62-1.19] | 0.357 | 0.926 [0.70-1.22] | 0.578 | 8.2 | Highly Robust |
| Rectus Abdominus Attenuation | 0.928 [0.69-1.24] | 0.615 | 0.908 [0.70-1.17] | 0.457 | -2.2 | Highly Robust |
| Subcutaneous Fat Index | 0.644 [0.39-1.07] | 0.09 | 0.636 [0.44-0.91] | 0.015 | -1.2 | Not Robust |
| Visceral Fat Index | 1.088 [0.78-1.52] | 0.624 | 1.062 [0.82-1.38] | 0.654 | -2.4 | Highly Robust |
| Subcutaneous Fat Attenuation | 1.838 [1.46-2.32] | <0.001 | 1.466 [1.21-1.78] | <0.001 | -20.2 | Highly Robust |
| Visceral Fat Attenuation | 1.498 [1.17-1.92] | 0.001 | 1.208 [0.98-1.48] | 0.07 | -19.4 | Not Robust |
